# A comparative evaluation of laser bandage and surgical stent as palatal donor site dressing after free gingival graft surgery: A randomized controlled clinical trial

**DOI:** 10.34172/japid.025.3380

**Published:** 2025-10-18

**Authors:** Pireethi Poonkundran, Rudrakshi Chickanna, Karthikeyan Bangalore Varadhan, Munivenkatappa Lakshmaiah Venkatesh Prabhuji

**Affiliations:** Department of Periodontology, Krishnadevaraya College of Dental Sciences, Bangalore, India

**Keywords:** Autograft, Charring, Free gingival graft, Laser bandage, Palatal donor site, Surgical stent

## Abstract

**Background.:**

The free gingival graft (FGG) harvesting technique creates an open wound that heals by secondary intention. Retarded wound healing, excessive bleeding, and postoperative pain have been reported as frequent complications. To overcome these problems, various products have been developed to heal the ailing site. Lasers can be considered a good choice for wound coverage of the donor site due to their effective tissue ablation, hemostatic, and bactericidal effects. The present randomized clinical trial was performed to evaluate the effectiveness of the diode laser bandage in achieving donor site hemostasis and compare wound healing with the surgical stent.

**Methods.:**

Twenty-four healthy individuals meeting the inclusion and exclusion criteria were recruited for the study. Following graft harvesting, the participants were assigned to the control (gel form hemostatic agent with surgical stent [GF+SS] [n=12]) or test (laser bandage [LB] [n=12]) groups. Clinical parameters, including pain (visual analog scale [VAS] score), bleeding, re-epithelialization, wound healing, color match, and number of analgesics consumed, were recorded at baseline and on the 7th, 14th, and 30th days. *P*<0.05 was set for statistical significance.

**Results.:**

Surgical procedures and postoperative sequelae were uneventful. VAS scores between the control and test groups were significant at baseline and on the 7th and 14th days. Parameters such as re- epithelialization, color match, and number of analgesics achieved statistically significant improvements.

**Conclusion.:**

Within the limitations of the present study, it can be concluded that the laser bandage is a better option for palatal wound protection following FGG harvesting.

## Introduction

 Autogenous soft tissue grafting has been increasingly used in clinical practice to augment tissue thickness, re-establish adequate width of keratinized tissue, correct mucogingival deformities, and improve aesthetics at tooth and dental implant sites.^1^ A soft tissue graft harvested from the palate with the overlying epithelium is defined as the free gingival graft (FGG).^2^ The disadvantages of harvesting a free gingival graft include increased discomfort and potential for postoperative bleeding from the donor area by virtue of a large wound that heals by secondary intention.^3^ The recuperation period following graft harvesting is long, and no definitive method has been suggested to decrease donor site morbidity.

 Manson^4^ suggested that a dressing is required to protect a palatal wound from trauma and oral fluids, thereby providing comfort, rapid healing, preventing the proliferation of granulation tissue, and controlling hemorrhage. In an effort to accelerate palatal donor site healing and reduce prolonged bleeding and pain caused by the palatal wound, hemostatic agents, including absorbable synthetic collagen, cyanoacrylate, oxidized regenerated cellulose, ferric subsulphate, and, more recently, platelet concentrate, have been used.^5,6^ However, these materials may cause adverse effects such as allergies, foreign body reactions, or retarded healing of the wound.

 The evolution of lasers over the past decade has been phenomenal, significantly altering the management of wounds following periodontal surgery. The unique characteristics of laser technology, such as ablation, hemostasis, bactericidal and detoxification effects, and promotion of tissue regeneration and wound healing, make it possible to treat soft and hard tissues. The diode laser can be used due to its ease of application and low cost, adequate coagulation, reduced inflammation and pain, improved repair and recovery, and rare postoperative complications.^7^

 Laser exhibits hemostatic effects due to its ‘hot-tip’ effect caused by heat accumulation at the end of the fiber. This produces a thick coagulation layer called a “laser bandage or biologic bandage.”^8^ Coleton placed a biologic bandage at the donor site using a CO_2_ laser, set at 5 W continuous wave in ablative mode. It is also referred to as “char layer” or “eschar” on the treated surface. The current study evaluated the effectiveness of diode laser bandage in achieving donor site hemostasis and compared wound healing with a standardized hemostatic agent and a surgical stent.

## Methods

 The present study was a prospective, randomized clinical trial (ClinicalTrials.gov identifier:NCT05841641), approved by the Ethics Committee (Ethical Comm/020/2020-21) of Krishnadevaraya College of Dental Sciences, affiliated to the Rajiv Gandhi University of Health Sciences and conducted in accordance with the ethical principles of the World Medical Association Declaration of Helsinki, version VI, 2002.

###  Study population

 Twenty-four participants were recruited from the Outpatient Section of the Department of Periodontology, Krishnadevaraya College of Dental Sciences, Bangalore, India, with a mean age of31.1 ± 5.53 years (Table 1).

 Patients who met the following inclusion criteria were included in the study: (1) systemically healthy subjects, non-smokers, and no record of allergies; (2) patients willing to participate in the study; (3) patients in the 25–55 years age group; (4) patients with esthetic concerns; (5) patients with a palatal mucosa thickness of > 2.5 mm; (6) a full-mouth plaque score (FMPS) of < 20% and a full-mouth bleeding score (FMBS) of < 20%. The exclusion criteria were: (1) patients with any systemic diseases; (2) patients with a palatal mucosa thickness of < 2.5 mm; (3) patients with a history of coagulation disorders; (4) pregnant and lactating females; (5) a history of tobacco usage; (6) patients taking medication that interferes with healing. All the patients received an explanation about the risks and benefits of the clinical procedures and signed a written informed consent form.

###  Study design and treatment protocols

**Figure 1:**
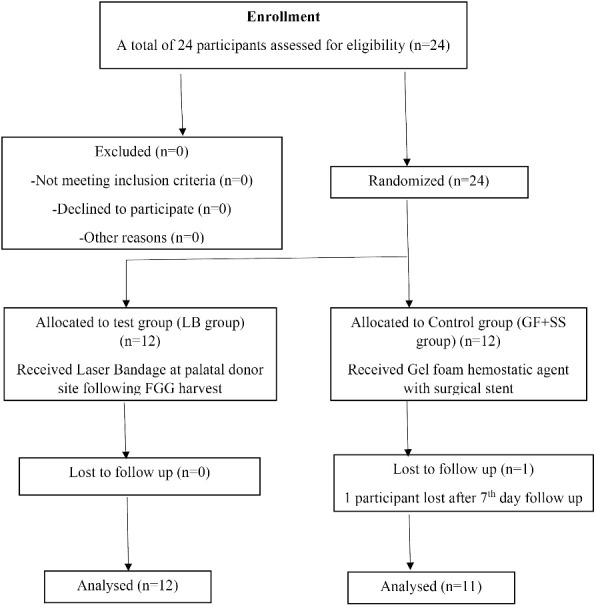
CONSORT flowchart

 In this unicenter randomized control trial, the participants (n = 24) were randomly assigned to the control (gel form hemostatic agent and surgical stent [GF + SS] or test (laser bandage [LB]) groups with a 1:1 allocation ratio based on a generated randomization scheme according to the Consolidated Standards of Reporting Trials (CONSORT) criteria, 2010 (Figure 1). All the patients’ clinical examinations were performed, and they received periodontal therapy. The examination included palatal mucosal thickness, FMPS, and FMBS.

###  Preoperative Procedures 

 All the patients enrolled in the study underwent a thorough scaling and root planning (SRP) procedure, followed by mouth rinses. The patients underwent a hemogram and were given oral hygiene instructions. After prophylaxis, SRP were performed when necessary, and the patients were enrolled into two groups:

- Control group (n = 12; GF + SS): Free gingival graft palatal donor site; gel-form hemostatic agent with surgical stent
- Test group (n = 12; LB): Free gingival graft palatal donor site; laser bandage.

###  Surgical procedure 

**Figure 2:**
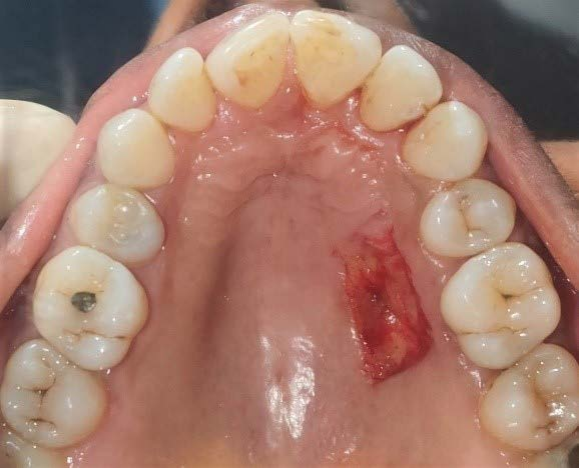
Donor site after FGG harvesting

 All the patients underwent the same surgical technique; to minimize variations in the surgical technique, all the surgical procedures were performed by one surgeon (PP). After a regional local anesthesia [2% lignocaine hydrochloride with 1:80,000 epinephrine] was injected around the greater palatine nerve, the FGG was harvested as follows. The donor site extended from the distal line angle of the canine to the mesial line angle of the maxillary first molar by a conventional scalpel (#15C, Swann Morton). A 1.5-mm split-thickness and rectangular gingival graft was obtained (Figure 2).

**Figure 3:**
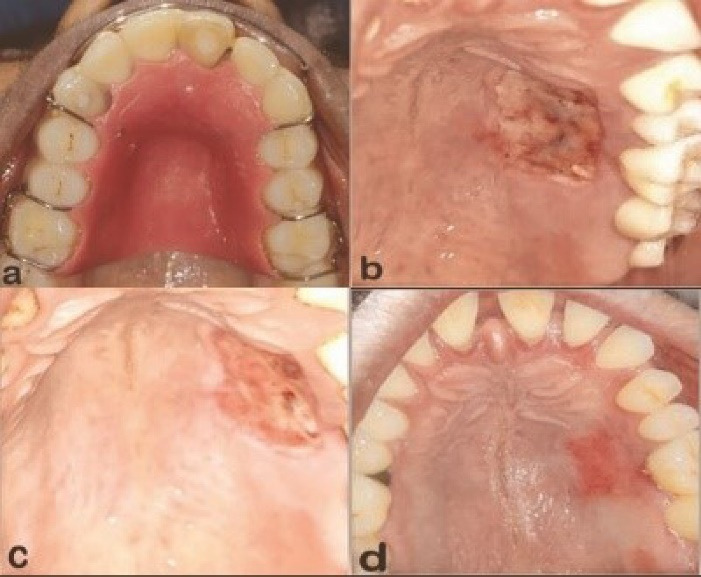
a) Donor site with palatal surgical stent (control group). b) 7th day postoperative control group. c) 14th day postoperative control group. d) 30th day postoperative control group

 After graft harvesting, the participants assigned to the control group (GF + SS) received a moist sterile gauze, which was placed over the palatal wound for 1 minute with moderate pressure. Pressure was applied to the wound to compress the hemostatic agent (AbGelTM), and the wound was sutured to achieve initial binding to the wound surface. Following this, a clear plastic palatal stent was placed over the wound, and the patient was instructed to wear the stent for a minimum of two days and up to seven days as needed for comfort (Figure 3a).

**Figure 4:**
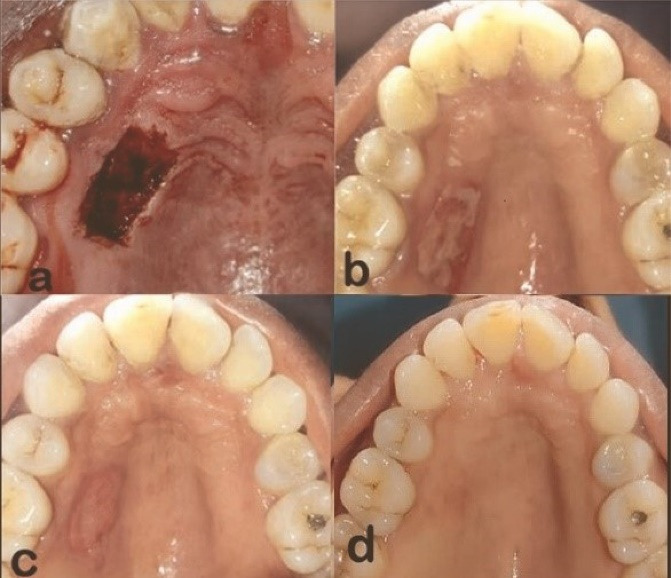
a) Laser bandage (LB) (test group). b) 7th day postoperative test group. c) 14th day postoperative test group. d) 30th day postoperative test group

 In the test group (LB), a gallium-aluminum-arsenide (Ga-AL-As) diode laser was used to create a biological bandage, at a wavelength of 810 nm and an intensity calibrated by the manufacturer (FOX – A.R.C. LASER). The laser was set at 5 W in continuous-wave mode. Laser energy was applied via a 400-μm optical fiber. The optical fiber was positioned perpendicularly in contact mode until the entire wound area was charred(Figure 4a).

**Table 1:** Gender and age distribution stratified by group

**Group**	**Gender (M/F)**	**Age**
Control (n = 12)	8/4	30.2 ± 5.1
Test (n = 12)	8/4	32.0 ± 6.01
*P* value	1.000	0.0451

**Table 2:** Comparison of mean VAS scores between the two groups at different time intervals

**Visit**	**Group**	**N**	**Mean**	**SD**	***P*** ** value**
Baseline	Control	12	9.8	0.622	0.001
	Test	12	7.8	1.528	
7th Day	Control	12	7.4	0.996	< 0.001
	Test	12	3.5	1.567	
14th Day	Control	11	4.6	1.206	< 0.001
	Test	12	0.3	0.622	
30th Day	Control	11	0.2	0.603	0.307

**Table 3:** Inter-group comparison of distribution of Landry Wound Healing Index scores at study intervals

**Wound healing**	**7th Day**				**14th Day**				**30th Day**			
	**Control**		**Test**		**Control**		**Test**		**Control**		**Test**	
Very poor	9	75.0%	0	0.0%	0	0.0%	0	0.0%	0	0.0%	0	0.0%
Poor	3	25.0%	1	8.3%	3	27.3%	0	0.0%	0	0.0%	0	0.0%
Good	0	0.0%	10	83.3%	7	63.6%	0	0.0%	1	9.1%	0	0.0%
Very good	0	0.0%	1	8.3%	1	9.1%	9	75.0%	5	45.5%	0	0.0%
Excellent	0	0.0%	0	0.0%	0	0.0%	3	25.0%	5	45.5%	12	100.0%
*P* value	< 0.001				< 0.001				0.012			

**Figure 5:**
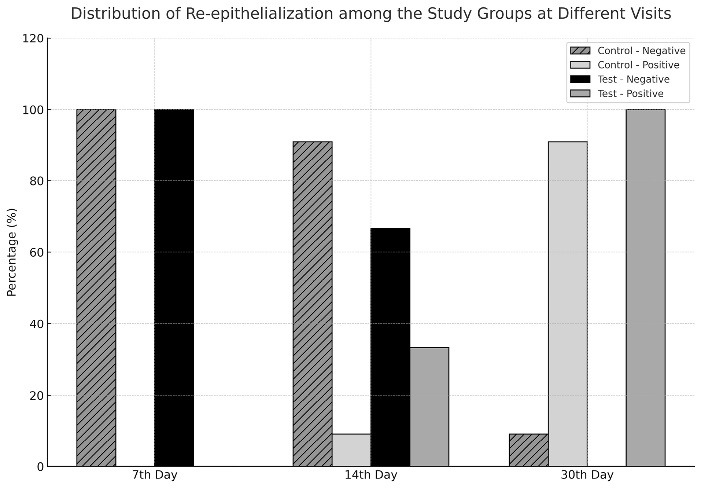
Distribution of re-epithelialization in the study groups at different visits

###  Postoperative care

 Postoperative instructions included 0.2% chlorhexidine gluconate mouth rinse three times daily for one minute and avoidance of brushing at the surgical site for two weeks. Postoperative pain and edema were controlled with non-steroidal anti-inflammatory drug (tab ibuprofen, 400 mg) and antibiotic amoxicillin, 500 mg, three times daily for three days after meals.

###  Postoperative evaluation

 Recall visits were scheduled on the 7th day (Figure 3b & 4b), 14th day (Figure 3c & 4c), and 30th day (Figure 3d and 4d) for a month to assess the wound healing and all the parameters of the control and test groups, respectively. Any complications, including soft-tissue changes in color, inflammation, and bleeding, were documented throughout the follow-up period.

###  Clinical measurements

 All the evaluations were made by one of the authors, who was blinded to the treatment assignment. In order to evaluate the healing process, clinical measures were collected as follows: (1) discomfort/pain, VAS score for pain, which ranged from 0 (no pain) to 10 (severe pain), represented by a continuous line measuring 10 cm in length, assessed at baseline, 7th, 14th, and 30th day; (2) to assess immediate and delayed bleeding, the patients were asked to report their postoperative bleeding as ‘bleeding present ( + )’ or ‘bleeding absent (-);’(3) wound healing assessment; the palatal wounds were scored using the Landry WHI at baseline and on the 7th, 14th, and 30th days; (4) wound epithelialization; re-epithelialization was evaluated clinically by the peroxide bubbling test; (5) color match; on the 7th, 14th, and 30th day, the color of the palatal mucosa was assessed by comparing it with that of the adjacent and opposite side by using Manchester Scar Score (MSS) (6); palatal tissue consistency; the consistency of the palatal mucosa was assessed on the 30th day by palpating with blunt instrument and was scored as soft or firm; (7) the number of analgesics; the patients were asked to record the number of analgesics taken for pain relief during the first seven postoperative days.

###  Statistical analysis

 Data were analyzed with SPSS 18.5 (SPSS for Windows). A descriptive analysis (mean, standard deviation, and frequency distribution) was conducted on the collected data.

 The Mann-Whitney testwas used to compare the incidence of immediate and delayed bleeding, based on Landry wound-healing index scores. Intergroup comparisons at different time intervals of color match (Manchester Scar Scale) and re-epithelialization (H_2_O_2_ bubble test) were performed using the chi-squared test. A *P* value of < 0.05 was considered statistically significant.

## Results

 The study began with 24 participants, but one participant in the control group dropped out after the second week of evaluation. The trial was ended upon completion of the 30-day follow-up visits, and the patient data were analyzed.

Table 1 presents the characteristics of the patient sample. The initial statistical analysis revealed no significant differences in age or gender between the groups at baseline.

Table 2 presents the data on VAS pain scores. During the 7th and 14th postoperative days, mean VAS pain scores were significantly higher in the control group (*P* ≤ 0.001). However, no significant difference was observed between the two groups on the 30th day. Six patients in the control group reported immediate palatal bleeding. The differences between the two groups were not statistically significant (*P* = 0.007). In the first 7 days postoperatively, neither the test nor the control group reported any delayed bleeding.

 Intragroup comparison of wound healing (LWHI) on the 7th, 14th, and 30th days in the test and control groups revealed statistically significant differences (*P* < 0.001). The LB group had better LWHI scores than the control group on the 7th and 14th days (*P* < 0.001). Also, the LWHI scores were significantly different on the 30th day (*P* = 0.027) (Table 3). None of the patients showed total re-epithelialization on the 7th day. On the 30th day, 100% of sites in the test group achieved complete re-epithelialization, compared with 90.9% in the control group. From the results of the H_2_O_2_ bubble test, we concluded that there was a significant relationship between the Laser bandage and re-epithelialization (*P* < 0.001) (Figure 5).

 MSS scores exhibiting color match between control and test on the 7th and 14th day showed statistically significant differences (*P* < 0.001). A subjective mismatch persisted in both groups on the 7th and 14th days. On the 30th day, a comparison between the control and test groups revealed statistically significant differences (*P* = 0.014), with perfect matches of 54.5% and 100% in the control and test groups, respectively. Tissue consistency did not differ significantly between the groups on the 30th day of the clinical follow-up visit (*P* = 0427).

 All the patients reported pain at the donor site following graft surgery, and the mean number of analgesics taken was 10.3 ± 1.875 in the control group, with 5.3 ± 1.215 in the test group.A statistically significant difference was found between the control and test groups (*P* < 0.001). More analgesics were taken to alleviate pain in the control group.

## Discussion

 The present prospective, randomized controlled clinical trial was conducted to evaluate the efficacy of the diode laser in creating a laser bandage and to assess wound healing and patient-centered outcomes. Although the literature indicates that lasers benefit oral wound healing, this is the first randomized clinical trial to investigate the ablative effects of the diode laser on wound healing.^9-11^

 Post-harvest healing of the palatal donor site wound is a complex process involving multiple cellular and biological processes.^12^ Donor site morbidities, including complications from postoperative pain and excessive bleeding, retard wound healing during the healing phase.^13,14^ An optimal method and technique to reduce patient morbidity and enhance wound healing in the palatal donor site have been developed. Laser therapy is also one of the modalities of palatal wound healing. Advantages include reduced postoperative pain, improved hemostasis, reduced bacterial population at the surgical site, and reduced need for suturing.

 Pick et al^15^ compared wound healing outcomes after scalpel, Nd:YAG laser, and electrosurgery in oral mucosa. An Nd:YAG laser was used in non-contact mode at extremely low power to create a biologic bandage from the patient’s own tissue. This study concluded that the Nd:YAG laser group experienced immediate pain relief and showed evidence that healing time may be significantly reduced. In the study, a diode laser was used in continuous, contact mode to create an ‘eschar’ of the wound area.

 Ustaoglu et al^16^ and Ozcelik et al^17^ studied the FGG donor site to assess palatal wound healing using Laser Therapy. VAS scores assessed at study intervals showed similar subjective assessments and statistically significant differences (*P* < 0.0001) between the control and test group. As in the present study, VAS scores were observed in both studies.

 Patients in our study were asked to report postoperative bleeding as present ( + ) or absent (-), and decreased postoperative morbidity after FGG harvesting was observed. Results assessing bleeding immediately postoperatively through the first 7 days showed a statistically significant difference (*P* = 0.007) between the test and control groups in the present study. In all the above-mentioned studies,^16,17^ significantly better VAS scores and bleeding might be due to the analgesic and acceleration effect of lasers. Contrary to our study, Heidari et al^18^ reported no significant difference in immediate or delayed bleeding, whereas immediate bleeding occurred right after low-powered laser irradiation in two cases.

 The variances for the Landry WHI were significantly different between the two groups. Higher scores were recorded for the laser bandage group. Dias et al^10^ reported positive effects of laser irradiation on the palatal donor site of CTG. These positive effects of laser bandage on wound healing were reported in another study that used LB following gingivectomy.^19^ Although the exact mechanism of action of lasers on palatal donor site wound healing after FGG harvesting is not clear, a recent clinical trial demonstrated that the level of TGF, PDGF-BB, and IL-8 in the palatal wound fluid increased after application of low-level lasers.^18^

 The rate of palatal wound epithelialization is determined by the relationship between the proliferative and migratory activity of peripheral keratinocytes and the collagen synthesis of the exposed tissue.^20^Ehab et al^21^ assessed epithelialization following FGG harvest using Alvogyl, an absorbable gelatin sponge, with the bubble test. Notable differences in healing between groups were seen at the fourth week. In contrast, the present study showed significant differences between groups at the second week (14th day). Compared with Ehab and colleagues’ study, our study demonstrated the superiority of laser treatment for the donor site.

 The evaluation of color match at each postoperative visit provided valuable insight into differences in wound healing between patients across treatment groups.^5^ Keceli et al^13^ assessed color match following graft procurement in their study on palatal donor site hemostasis and wound healing using a medicinal plant extract. The authors concluded that color match was slightly better (*P* < 0.05) in their test group, a result similar to ours.

 Diode laser has been used to cut or vaporize soft tissue in continuous or gated-pulse modes in a contact mode. Thermal necrosis of < 1 mm can be achieved to provide adequate surgical precision and hemostasis for soft tissue procedures.^8^ In the present study, a Ga-Al-As diode laser was used to treat the wound area, which had the advantages of less need for analgesics and eliminated the need for sutures. Despite the positive results presented in this study, caution should be exercised while using the laser. When a laser with an energy of 5 W is used, care must be taken to prevent thermal damage to the underlying periosteum and bone. More well-designed randomized clinical trials with larger sample sizes must be conducted in this area to identify the optimal laser irradiation parameters that promote healing while reducing patients’ discomfort.

## Conclusion

 Within the limitations of the present prospective study, it can be concluded that the laser bandage is undoubtedly a better option for palatal wound protection following FGG harvesting. This technique offers greater advantages, including better wound healing, a simpler execution, less trauma, faster hemostasis, and minimal postoperative complications, compared to other healing techniques. Furthermore, laser bandage can be easily recommended in a variety of clinical situations where suturing is complicated and in other secondary wound healing situations.

## Competing Interests

 The authors declare that they have no competing interests.

## Data Availability

 All data regarding the methodology of the manuscript has been shared.

## Ethical Approval

 The present study (ClinicalTrials.gov identifier:NCT05841641) was approved by the Ethics Committee (Ethical Comm/ 020 /2020-21) of Krishnadevaraya College of Dental Sciences, affiliated to the Rajiv Gandhi University of Health Sciences.
